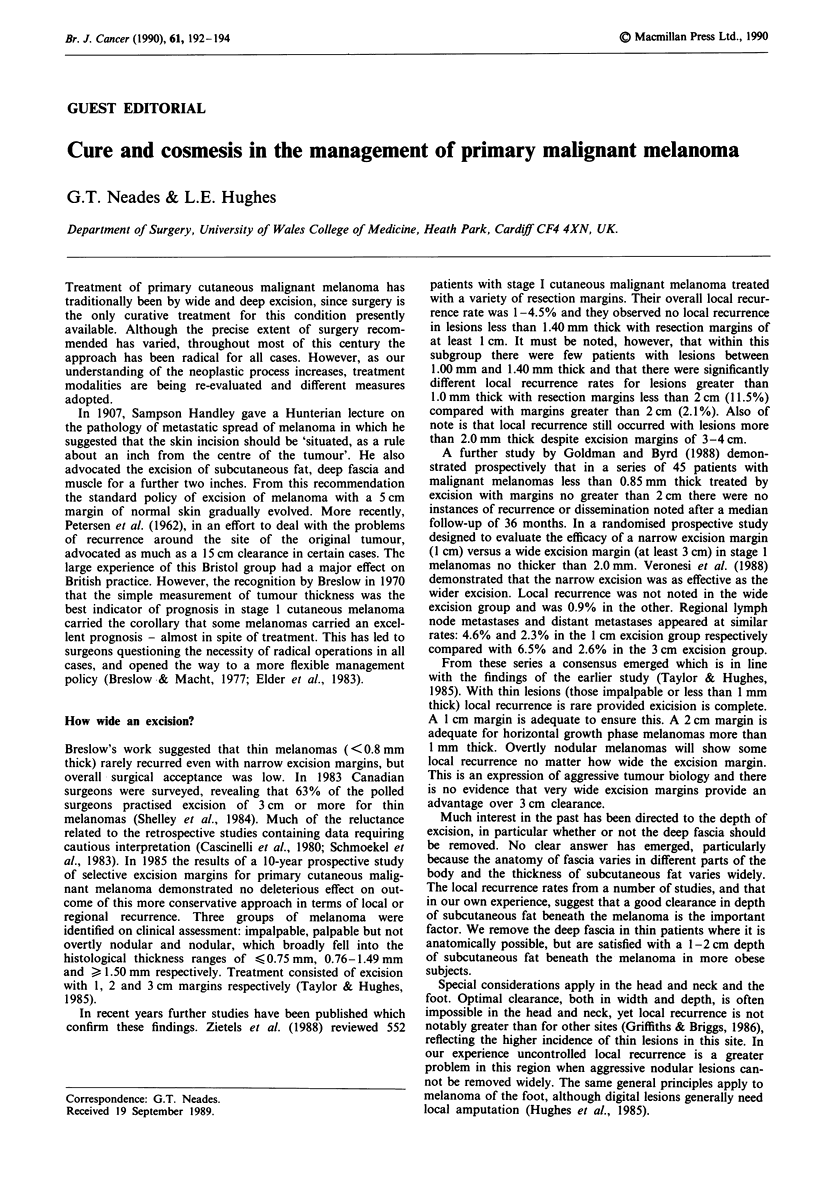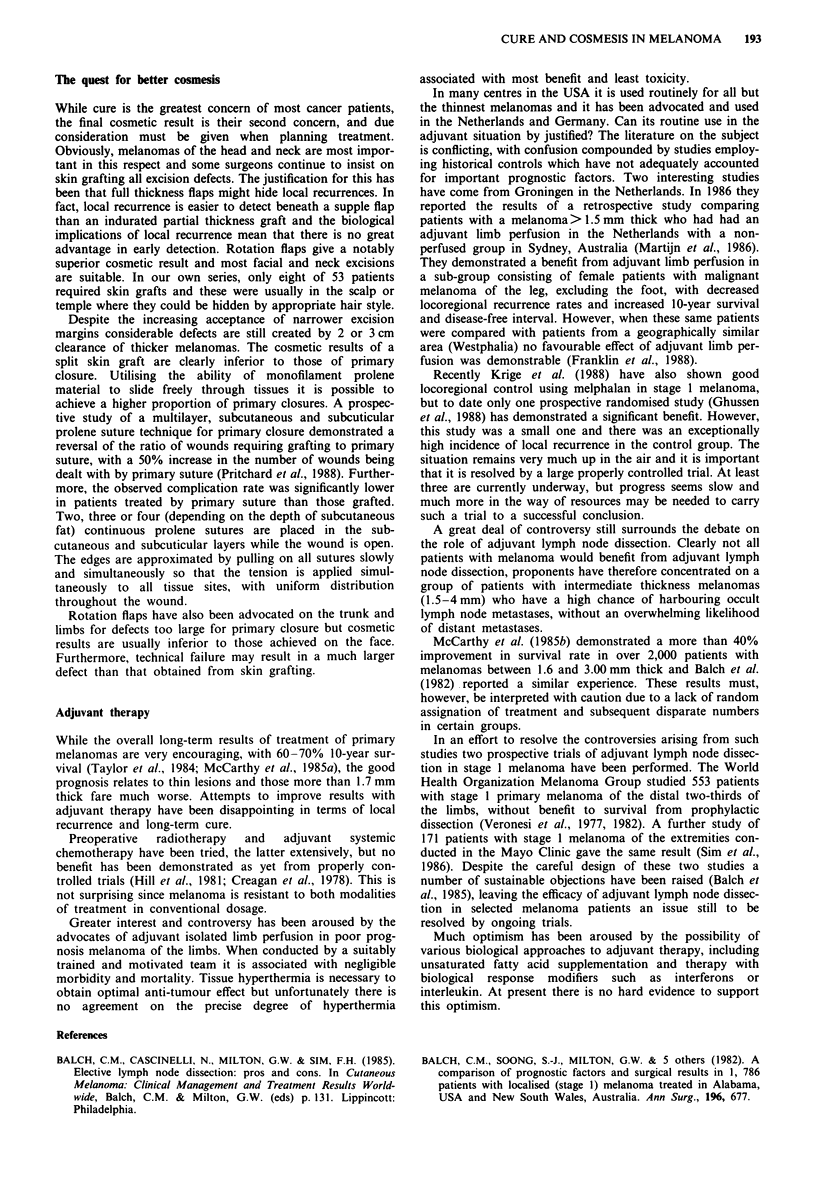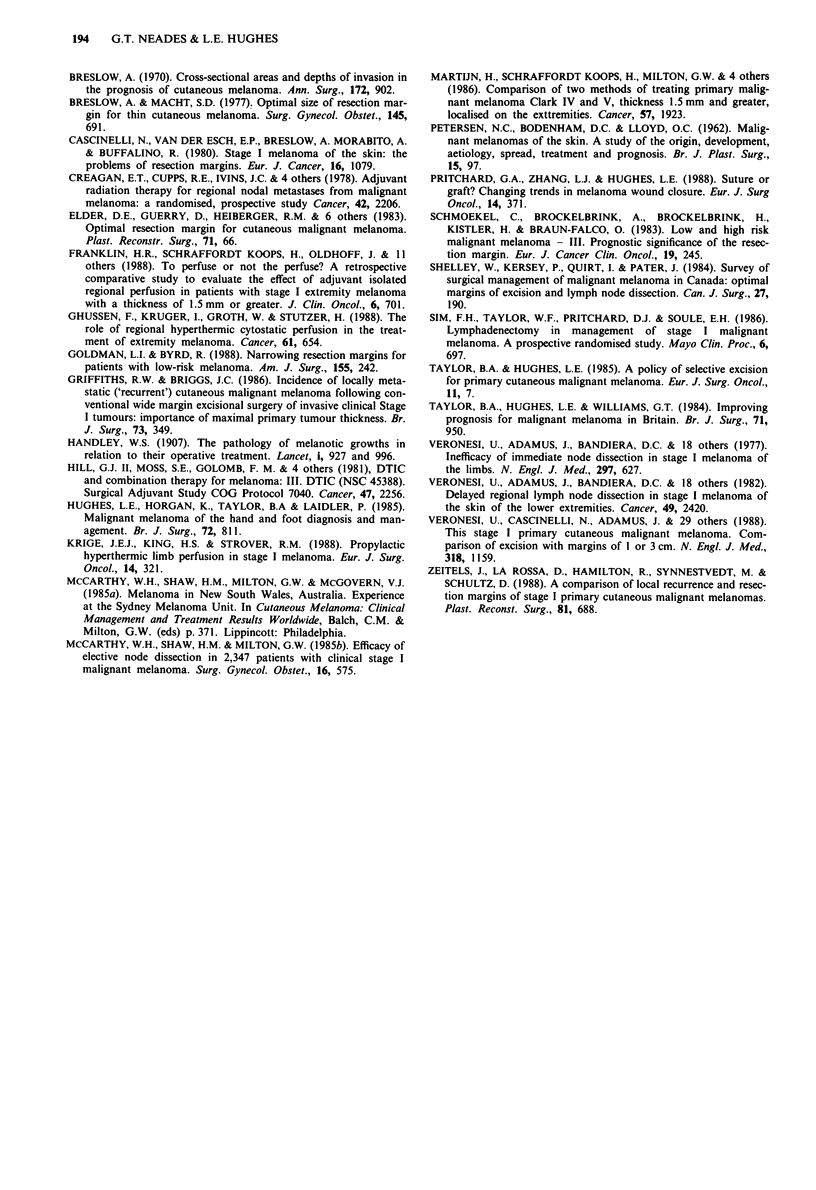# Cure and cosmesis in the management of primary malignant melanoma.

**DOI:** 10.1038/bjc.1990.36

**Published:** 1990-02

**Authors:** G. T. Neades, L. E. Hughes

**Affiliations:** Department of Surgery, University of Wales College of Medicine, Heath Park, Cardiff, UK.


*Br. J. Cancer* (1990), **61**, 192–194 © Macmillan Press Ltd., 1990

GUEST EDITORIAL

### Cure and cosmesis in the management of primary malignant melanoma

G.T. Neades & L.E. Hughes

*Department of Surgery, University of Wales College of Medicine, Heath Park, Cardiff CF4 4XN, UK.*

Treatment of primary cutaneous malignant melanoma has traditionally been by wide and deep excision, since surgery is the only curative treatment for this condition presently available. Although the precise extent of surgery recommended has varied, throughout most of this century the approach has been radical for all cases. However, as our understanding of the neoplastic process increases, treatment modalities are being re-evaluated and different measures adopted.

In 1907, Sampson Handley gave a Hunterian lecture on the pathology of metastatic spread of melanoma in which he suggested that the skin incision should be 'situated, as a rule about an inch from the centre of the tumour'. He also advocated the excision of subcutaneous fat, deep fascia and muscle for a further two inches. From this recommendation the standard policy of excision of melanoma with a 5 cm margin of normal skin gradually evolved. More recently, Petersen *et al.* (1962), in an effort to deal with the problems of recurrence around the site of the original tumour, advocated as much as a 15 cm clearance in certain cases. The large experience of this Bristol group had a major effect on British practice. However, the recognition by Breslow in 1970 that the simple measurement of tumour thickness was the best indicator of prognosis in stage 1 cutaneous melanoma carried the corollary that some melanomas carried an excellent prognosis – almost in spite of treatment. This has led to surgeons questioning the necessity of radical operations in all cases, and opened the way to a more flexible management policy (Breslow & Macht, 1977; Elder *et al.*, 1983).

### How wide an excision?

Breslow's work suggested that thin melanomas (<0.8 mm thick) rarely recurred even with narrow excision margins, but overall surgical acceptance was low. In 1983 Canadian surgeons were surveyed, revealing that 63% of the polled surgeons practised excision of 3 cm or more for thin melanomas (Shelley *et al.*, 1984). Much of the reluctance related to the retrospective studies containing data requiring cautious interpretation (Cascinelli *et al.*, 1980; Schmoekel *et al.*, 1983). In 1985 the results of a 10-year prospective study of selective excision margins for primary cutaneous malignant melanoma demonstrated no deleterious effect on outcome of this more conservative approach in terms of local or regional recurrence. Three groups of melanoma were identified on clinical assessment: impalpable, palpable but not overtly nodular and nodular, which broadly fell into the histological thickness ranges of ⩽0.75 mm, 0.76–1.49 mm and ⩾1.50 mm respectively. Treatment consisted of excision with 1, 2 and 3 cm margins respectively (Taylor & Hughes, 1985).

In recent years further studies have been published which confirm these findings. Zietels *et al.* (1988) reviewed 552 patients with stage I cutaneous malignant melanoma treated with a variety of resection margins. Their overall local recurrence rate was 1–4.5% and they observed no local recurrence in lesions less than 1.40 mm thick with resection margins of at least 1 cm. It must be noted, however, that within this subgroup there were few patients with lesions between 1.00 mm and 1.40 mm thick and that there were significantly different local recurrence rates for lesions greater than 1.0 mm thick with resection margins less than 2 cm (11.5%) compared with margins greater than 2 cm (2.1%). Also of note is that local recurrence still occurred with lesions more than 2.0 mm thick despite excision margins of 3–4 cm.

A further study by Goldman and Byrd (1988) demonstrated prospectively that in a series of 45 patients with malignant melanomas less than 0.85 mm thick treated by excision with margins no greater than 2 cm there were no instances of recurrence or dissemination noted after a median follow-up of 36 months. In a randomised prospective study designed to evaluate the efficacy of a narrow excision margin (1 cm) versus a wide excision margin (at least 3 cm) in stage 1 melanomas no thicker than 2.0 mm. Veronesi *et al.* (1988) demonstrated that the narrow excision was as effective as the wider excision. Local recurrence was not noted in the wide excision group and was 0.9% in the other. Regional lymph node metastases and distant metastases appeared at similar rates: 4.6% and 2.3% in the 1 cm excision group respectively compared with 6.5% and 2.6% in the 3 cm excision group.

From these series a consensus emerged which is in line with the findings of the earlier study (Taylor & Hughes, 1985). With thin lesions (those impalpable or less than 1 mm thick) local recurrence is rare provided exicision is complete. A 1 cm margin is adequate to ensure this. A 2 cm margin is adequate for horizontal growth phase melanomas more than 1 mm thick. Overtly nodular melanomas will show some local recurrence no matter how wide the excision margin. This is an expression of aggressive tumour biology and there is no evidence that very wide excision margins provide an advantage over 3 cm clearance.

Much interest in the past has been directed to the depth of excision, in particular whether or not the deep fascia should be removed. No clear answer has emerged, particularly because the anatomy of fascia varies in different parts of the body and the thickness of subcutaneous fat varies widely. The local recurrence rates from a number of studies, and that in our own experience, suggest that a good clearance in depth of subcutaneous fat beneath the melanoma is the important factor. We remove the deep fascia in thin patients where it is anatomically possible, but are satisfied with a 1–2 cm depth of subcutaneous fat beneath the melanoma in more obese subjects.

Special considerations apply in the head and neck and the foot. Optimal clearance, both in width and depth, is often impossible in the head and neck, yet local recurrence is not notably greater than for other sites (Griffiths & Briggs, 1986), reflecting the higher incidence of thin lesions in this site. In our experience uncontrolled local recurrence is a greater problem in this region when aggressive nodular lesions cannot be removed widely. The same general principles apply to melanoma of the foot, although digital lesions generally need local amputation (Hughes *et al.*, 1985).

Correspondence: G.T. Neades.
Received 19 September 1989.

**The quest for better cosmesis**

While cure is the greatest concern of most cancer patients, the final cosmetic result is their second concern, and due consideration must be given when planning treatment. Obviously, melanomas of the head and neck are most important in this respect and some surgeons continue to insist on skin grafting all excision defects. The justification for this has been that full thickness flaps might hide local recurrences. In fact, local recurrence is easier to detect beneath a supple flap than an indurated partial thickness graft and the biological implications of local recurrence mean that there is no great advantage in early detection. Rotation flaps give a notably superior cosmetic result and most facial and neck excisions are suitable. In our own series, only eight of 53 patients required skin grafts and these were usually in the scalp or temple where they could be hidden by appropriate hair style.

Despite the increasing acceptance of narrower excision margins considerable defects are still created by 2 or 3 cm clearance of thicker melanomas. The cosmetic results of a split skin graft are clearly inferior to those of primary closure. Utilising the ability of monofilament prolene material to slide freely through tissues it is possible to achieve a higher proportion of primary closures. A prospective study of a multilayer, subcutaneous and subcuticular prolene suture technique for primary closure demonstrated a reversal of the ratio of wounds requiring grafting to primary suture, with a 50% increase in the number of wounds being dealt with by primary suture (Pritchard *et al.*, 1988). Furthermore, the observed complication rate was significantly lower in patients treated by primary suture than those grafted. Two, three or four (depending on the depth of subcutaneous fat) continuous prolene sutures are placed in the subcutaneous and subcuticular layers while the wound is open. The edges are approximated by pulling on all sutures slowly and simultaneously so that the tension is applied simultaneously to all tissue sites, with uniform distribution throughout the wound.

Rotation flaps have also been advocated on the trunk and limbs for defects too large for primary closure but cosmetic results are usually inferior to those achieved on the face. Furthermore, technical failure may result in a much larger defect than that obtained from skin grafting.

**Adjuvant therapy**

While the overall long-term results of treatment of primary melanomas are very encouraging, with 60–70% 10-year survival (Taylor *et al.*, 1984; McCarthy *et al.*, 1985*a*), the good prognosis relates to thin lesions and those more than 1.7 mm thick fare much worse. Attempts to improve results with adjuvant therapy have been disappointing in terms of local recurrence and long-term cure.

Preoperative radiotherapy and adjuvant systemic chemotherapy have been tried, the latter extensively, but no benefit has been demonstrated as yet from properly controlled trials (Hill *et al.*, 1981; Creagan *et al.*, 1978). This is not surprising since melanoma is resistant to both modalities of treatment in conventional dosage.

Greater interest and controversy has been aroused by the advocates of adjuvant isolated limb perfusion in poor prognosis melanoma of the limbs. When conducted by a suitably trained and motivated team it is associated with negligible morbidity and mortality. Tissue hyperthermia is necessary to obtain optimal anti-tumour effect but unfortunately there is no agreement on the precise degree of hyperthermia associated with most benefit and least toxicity.

In many centres in the USA it is used routinely for all but the thinnest melanomas and it has been advocated and used in the Netherlands and Germany. Can its routine use in the adjuvant situation by justified? The literature on the subject is conflicting, with confusion compounded by studies employing historical controls which have not adequately accounted for important prognostic factors. Two interesting studies have come from Groningen in the Netherlands. In 1986 they reported the results of a retrospective study comparing patients with a melanoma > 1.5 mm thick who had had an adjuvant limb perfusion in the Netherlands with a non-perfused group in Sydney, Australia (Martijn *et al.*, 1986). They demonstrated a benefit from adjuvant limb perfusion in a sub-group consisting of female patients with malignant melanoma of the leg, excluding the foot, with decreased locoregional recurrence rates and increased 10-year survival and disease-free interval. However, when these same patients were compared with patients from a geographically similar area (Westphalia) no favourable effect of adjuvant limb perfusion was demonstrable (Franklin *et al.*, 1988).

Recently Krige *et al.* (1988) have also shown good locoregional control using melphalan in stage 1 melanoma, but to date only one prospective randomised study (Ghussen *et al.*, 1988) has demonstrated a significant benefit. However, this study was a small one and there was an exceptionally high incidence of local recurrence in the control group. The situation remains very much up in the air and it is important that it is resolved by a large properly controlled trial. At least three are currently underway, but progress seems slow and much more in the way of resources may be needed to carry such a trial to a successful conclusion.

A great deal of controversy still surrounds the debate on the role of adjuvant lymph node dissection. Clearly not all patients with melanoma would benefit from adjuvant lymph node dissection, proponents have therefore concentrated on a group of patients with intermediate thickness melanomas (1.5–4 mm) who have a high chance of harbouring occult lymph node metastases, without an overwhelming likelihood of distant metastases.

McCarthy *et al.* (1985*b*) demonstrated a more than 40% improvement in survival rate in over 2,000 patients with melanomas between 1.6 and 3.00 mm thick and Balch *et al.* (1982) reported a similar experience. These results must, however, be interpreted with caution due to a lack of random assignation of treatment and subsequent disparate numbers in certain groups.

In an effort to resolve the controversies arising from such studies two prospective trials of adjuvant lymph node dissection in stage 1 melanoma have been performed. The World Health Organization Melanoma Group studied 553 patients with stage 1 primary melanoma of the distal two-thirds of the limbs, without benefit to survival from prophylactic dissection (Veronesi *et al.*, 1977, 1982). A further study of 171 patients with stage 1 melanoma of the extremities conducted in the Mayo Clinic gave the same result (Sim *et al.*, 1986). Despite the careful design of these two studies a number of sustainable objections have been raised (Balch *et al.*, 1985), leaving the efficacy of adjuvant lymph node dissection in selected melanoma patients an issue still to be resolved by ongoing trials.

Much optimism has been aroused by the possibility of various biological approaches to adjuvant therapy, including unsaturated fatty acid supplementation and therapy with biological response modifiers such as interferons or interleukin. At present there is no hard evidence to support this optimism.

**References**

BALCH, C.M., CASCINELLI, N., MILTON, G.W. & SIM, F.H. (1985). Elective lymph node dissection: pros and cons. In *Cutaneous Melanoma: Clinical Management and Treatment Results Worldwide*, Balch, C.M. & Milton, G.W. (eds) p. 131. Lippincott: Philadelphia.

BALCH, C.M., SOONG, S.-J., MILTON, G.W. & 5 others (1982). A comparison of prognostic factors and surgical results in 1, 786 patients with localised (stage 1) melanoma treated in Alabama, USA and New South Wales, Australia. *Ann Surg.*, **196**, 677.

BRESLOW, A. (1970). Cross-sectional areas and depths of invasion in the prognosis of cutaneous melanoma. *Ann. Surg.*, **172,** 902.

BRESLOW, A. & MACHT, S.D. (1977). Optimal size of resection margin for thin cutaneous melanoma. *Surg. Gynecol. Obstet.*, **145,** 691.

CASCINELLI, N., VAN DER ESCH, E.P., BRESLOW, A. MORABITO, A. & BUFFALINO, R. (1980). Stage I melanoma of the skin: the problems of resection margins. *Eur. J. Cancer*, **16,** 1079.

CREAGAN, E.T., CUPPS, R.E., IVINS, J.C. & 4 others (1978). Adjuvant radiation therapy for regional nodal metastases from malignant melanoma: a randomised, prospective study *Cancer*, **42,** 2206.

ELDER, D.E., GUERRY, D., HEIBERGER, R.M. & 6 others (1983). Optimal resection margin for cutaneous malignant melanoma. *Plast. Reconstr. Surg.*, **71,** 66.

FRANKLIN, H.R., SCHRAFFORDT KOOPS, H., OLDHOFF, J. & 11 others (1988). To perfuse or not the perfuse? A retrospective comparative study to evaluate the effect of adjuvant isolated regional perfusion in patients with stage I extremity melanoma with a thickness of 1.5 mm or greater. *J. Clin. Oncol.*, **6,** 701.

GHUSSEN, F., KRUGER, I., GROTH, W. & STUTZER, H. (1988). The role of regional hyperthermic cytostatic perfusion in the treatment of extremity melanoma. *Cancer*, **61,** 654.

GOLDMAN, L.I. & BYRD, R. (1988). Narrowing resection margins for patients with low-risk melanoma. *Am. J. Surg.*, **155,** 242.

GRIFFITHS, R.W. & BRIGGS, J.C. (1986). Incidence of locally metastatic ('recurrent') cutaneous malignant melanoma following conventional wide margin excisional surgery of invasive clinical Stage I tumours: importance of maximal primary tumour thickness. *Br. J. Surg.*, **73,** 349.

HANDLEY, W.S. (1907). The pathology of melanotic growths in relation to their operative treatment. *Lancet*, **i,** 927 and 996.

HILL, G.J. II, MOSS, S.E., GOLOMB, F. M. & 4 others (1981), DTIC and combination therapy for melanoma: III. DTIC (NSC 45388). Surgical Adjuvant Study COG Protocol 7040. *Cancer*, **47,** 2256.

HUGHES, L.E., HORGAN, K., TAYLOR, B.A & LAIDLER, P. (1985). Malignant melanoma of the hand and foot diagnosis and management. *Br. J. Surg.*, **72,** 811.

KRIGE, J.E.J., KING, H.S. & STROVER, R.M. (1988). Propylactic hyperthermic limb perfusion in stage I melanoma. *Eur. J. Surg. Oncol.*, **14,** 321.

McCARTHY, W.H., SHAW, H.M., MILTON, G.W. & McGOVERN, V.J. (1985*a*). Melanoma in New South Wales, Australia. Experience at the Sydney Melanoma Unit. In *Cutaneous Melanoma: Clinical Management and Treatment Results Worldwide*, Balch, C.M. & Milton, G.W. (eds) p. 371. Lippincott: Philadelphia.

McCARTHY, W.H., SHAW, H.M. & MILTON, G.W. (1985*b*). Efficacy of elective node dissection in 2,347 patients with clinical stage I malignant melanoma. *Surg. Gynecol. Obstet.*, **16,** 575.

MARTIJN, H., SCHRAFFORDT KOOPS, H., MILTON, G.W. & 4 others (1986). Comparison of two methods of treating primary malignant melanoma Clark IV and V, thickness 1.5 mm and greater, localised on the exttremities. *Cancer*, **57,** 1923.

PETERSEN, N.C., BODENHAM, D.C. & LLOYD, O.C. (1962). Malignant melanomas of the skin. A study of the origin, development, aetiology, spread, treatment and prognosis. *Br. J. Plast. Surg.*, **15,** 97.

PRITCHARD, G.A., ZHANG, L.J. & HUGHES, L.E. (1988). Suture or graft? Changing trends in melanoma wound closure. *Eur. J. Surg Oncol.*, **14,** 371.

SCHMOEKEL, C., BROCKELBRINK, A., BROCKELBRINK, H., KISTLER, H. & BRAUN-FALCO, O. (1983). Low and high risk malignant melanoma – III. Prognostic significance of the resection margin. *Eur. J. Cancer Clin. Oncol.*, **19,** 245.

SHELLEY, W., KERSEY, P., QUIRT, I. & PATER, J. (1984). Survey of surgical management of malignant melanoma in Canada: optimal margins of excision and lymph node dissection. *Can. J. Surg.*, **27,** 190.

SIM, F.H., TAYLOR, W.F., PRITCHARD, D.J. & SOULE, E.H. (1986). Lymphadenectomy in management of stage I malignant melanoma. A prospective randomised study. *Mayo Clin. Proc.*, **6,** 697.

TAYLOR, B.A. & HUGHES, L.E. (1985). A policy of selective excision for primary cutaneous malignant melanoma. *Eur. J. Surg. Oncol.*, **11,** 7.

TAYLOR, B.A., HUGHES, L.E. & WILLIAMS, G.T. (1984). Improving prognosis for malignant melanoma in Britain. *Br. J. Surg.*, **71,** 950.

VERONESI, U., ADAMUS, J., BANDIERA, D.C. & 18 others (1977). Inefficacy of immediate node dissection in stage I melanoma of the limbs. *N. Engl. J. Med.*, **297,** 627.

VERONESI, U., ADAMUS, J., BANDIERA, D.C. & 18 others (1982). Delayed regional lymph node dissection in stage I melanoma of the skin of the lower extremities. *Cancer*, **49,** 2420.

VERONESI, U., CASCINELLI, N., ADAMUS, J. & 29 others (1988). This stage I primary cutaneous malignant melanoma. Comparison of excision with margins of 1 or 3 cm. *N. Engl. J. Med.*, **318,** 1159.

ZEITELS, J., LA ROSSA, D., HAMILTON, R., SYNNESTVEDT, M. & SCHULTZ, D. (1988). A comparison of local recurrence and resection margins of stage I primary cutaneous malignant melanomas. *Plast. Reconst. Surg.*, **81,** 688.